# Unmasking patient diversity: Exploring cognitive and antidepressive effects of electroconvulsive therapy

**DOI:** 10.1192/j.eurpsy.2024.1

**Published:** 2024-01-12

**Authors:** Kjersti Sellevåg, Christoffer A. Bartz-Johannessen, Ketil J. Oedegaard, Axel Nordenskjöld, Christine Mohn, Jeanette S. Bjørke, Ute Kessler

**Affiliations:** 1 NKS Olaviken Gerontopsychiatric Hospital, Askøy, Norway; 2 Department of Clinical Medicine, University of Bergen, Bergen, Norway; 3 Department of Psychiatry, Haukeland University Hospital, Bergen, Norway; 4 Norwegian Centre for Mental Disorders Research (NORMENT), Division of Mental Health and Addiction, Oslo University Hospital, Oslo, Norway; 5 The University Health Care Research Centre, Faculty of Medicine and Health, Örebro University, Örebro, Sweden; 6 National Centre for Suicide Research and Prevention (NSSF), Department of Clinical Medicine, University of Oslo, Oslo, Norway; 7 Psychiatric Division, Stavanger University Hospital, Stavanger, Norway

**Keywords:** Psychiatry, Depression, Neurostimulation treatment, Electroconvulsive therapy, ECT effectiveness, ECT cognitive outcomes

## Abstract

**Background:**

Electroconvulsive therapy (ECT) is an established treatment for depression, but more data on effectiveness and safety in clinical practice is needed. The aim of this register-based study was to investigate short-term effectiveness and cognitive safety after ECT, evaluated by clinicians and patients. Secondary, we investigated predictors for remission and cognitive decline.

**Methods:**

The study included 392 patients from the Regional Register for Neurostimulation Treatment in Western Norway. Depressive symptoms and cognitive function were assessed with Montgomery-Åsberg Depression Rating Scale and Mini-Mental State Examination (clinician-rated) and Beck Depression Inventory and Everyday Memory Questionnaire (patient-rated). Assessments were done prior to ECT-series and a mean of 1.7 days after (range 6 days before and 12 days after) end of ECT-series. Paired samples *t*-tests were extended by detailed, clinically relevant subgroups. Predictors were examined using logistic regression.

**Results:**

Clinician- and patient-rated remission rates were 49.5 and 41.0%, respectively. There was a large reduction in depressive symptoms and a small improvement in cognition after ECT, but we also identified subgroups with non-response of ECT in combination with cognitive decline (4.6% clinician-rated, 15.7% patient-rated). Positive predictors for patient- and clinician-rated remission were increasing age, shorter duration of depressive episode, and psychotic features. Antipsychotic medication at the commencement of treatment and previous ECT-treatment gave higher odds of clinician-rated remission, whereas higher pretreatment subjective depression level was associated with lower odds for patient-rated remission. Clinician-rated cognitive decline was predicted by higher pretreatment MMSE scores, whereas psychotic features, increasing age, and greater pretreatment subjective memory concerns were associated with lower odds for patient-rated cognitive decline.

**Conclusions:**

Our study supports ECT as an effective and safe treatment, although subgroups have a less favorable outcome. ECT should be considered at an early stage for older patients suffering from depression with psychotic features. Providing comprehensive and balanced information from clinicians and patients perspectives on effects and side effects, may assist in a joint consent process.

## Introduction

Depression is a prevalent [1] and disabling [2] disorder. Electroconvulsive therapy (ECT) is proven an effective [3, 4] and safe [5–8] treatment for severe or treatment-resistant depression. Despite this, its place in treatment algorithms varies. Several meta-analyses have assessed the efficacy of ECT, finding it more effective than placebo or antidepressant medication [3, 9, 10]. The included studies, however, are relatively old and not up to today’s methodological standards, and limited by differences in ECT technique, such as using the now outdated sine waveform. Later comparative effectiveness studies confirm ECT’s superiority over medication [11], and treatment modalities like repetitive transcranial magnetic stimulation [12] and transcranial direct current stimulation [13].

Despite advances in ECT technique aimed to minimize adverse effects, fear of cognitive side effects is an important reason for patients refraining from the treatment [14]. Several studies have shown transient negative cognitive effects [15, 16], and even improvements in the longer term [17, 18]. However, given the small number of patients included in relevant studies, heterogeneity in treatment parameters and assessment scales, and a discrepancy between objective and subjective cognitive measures [19], the conclusions should be interpreted with caution and more studies are warranted [18, 20]. The efficacy and safety of ECT in clinical trials may also differ from those in a real-world setting, due to differences in patient selection, treatment protocols, and clinical practice [21, 22]. It is thus important to investigate further the effectiveness and side effects in unselected patient cohorts. Several papers have reported either the antidepressive effects [21, 23, 24] or the cognitive side effects [15, 16] after ECT, but we are not aware of papers presenting a combination of the two in a four-quadrant model or a more detailed matrix. Identifying patient factors that predict outcome can help clinicians select the patients most likely to benefit from the treatment, and be of assistance for clinicians, patients, and their families in an informed, shared decision-making process.

The aim of this study was to investigate short-term effects of ECT on depression level and cognitive function in unipolar and bipolar depression, as evaluated by clinicians and by the patients themselves. Secondary, we wanted to identify predictors for clinician and patient-rated remission and cognitive decline.

## Methods

### Setting and sample

In this register-based cohort study, data were derived from the Regional Register for Neurostimulation Treatment in Western Norway [25]. The register was established in 2013 and includes patients from two ECT units; Stavanger University Hospital and Haukeland University Hospital. The units have similar demography and criteria for recommending ECT, based on national guidelines [26]. Norwegian mental health services are based on catchment areas, are publicly funded and equally available for everyone. Approximately 85% of the total patient population provided their consent to inclusion in the register. In the current study, patients with either unipolar (F32.1–F32.3) or bipolar (F31.1–F31.5) depressive episode or recurrent depressive disorder (F33.1–F33.3) were included between June 2013 and December 2021. If patients had more than one ECT-series in the register, only the first series was included in the study.

### Electroconvulsive therapy

ECT was administered with the Thymatron System IV (Somatics, Lake Bluff, IL). The preferred anesthetic was thiopental (mean dose 3.4 mg/kg, SD 1.0). If indicated, propofol (mean dose 1.9 mg/kg, SD 0.8), or etomidate (mean dose 0.2 mg/kg, SD 0.4) could be used. Further, succinylcholine (mean dose 1.0 mg/kg, SD 0.2) was administered. Electric stimulus was typically provided about 90s after administering the neuromuscular blockade. Patients were hyperoxygenated prior to the electric stimulation. The preferred application of stimulus was via right unilateral (RUL) electrode placement (97.7% of treatments) with a pulse width of 0.5 ms. The initial stimulus energy was determined by an age-based method [27]. If indications of an insufficient seizure or unsatisfactory effect on depressive symptoms, adjustments were considered, usually by increasing the electrical dose or switching electrode placement from uni- to bilateral (BL). If side effects such as cognitive decline were observed, alterations such as increasing the interval between treatments, lowering the electrical dose, changing electrode placement (from BL to RUL), or the use of ultra-brief pulse width (0.25 ms), were considered. The treatment was normally terminated if the patient obtained remission, experienced unacceptable side effects, or if further improvement was not expected.

### Diagnosis and assessments

Patient characteristics were extracted from the clinical charts by staff at the ECT-units, and from interviews with the patients. The severity of symptoms and cognitive functioning were assessed by treating clinicians (psychiatrists, psychologists, or psychiatric nurses), and by the patients themselves. Due to the nature of registry studies, the time of assessment pretreatment could vary from those included at referral to assessments done at the commencement of treatment. For the same reason, although assessments posttreatment preferably were done shortly after the last treatment, there was a span between a maximum of 6 days before and 12 days after (mean 1.7, SD 3.6 after) the last session. Psychotropic drugs were registered in relation to the first treatment session. This could differ from medication on time of referral due to factors such as tapering medication affecting seizure threshold (ST).

### Clinician-rated outcome measures

The severity of depressive symptoms was assessed using the Montgomery-Åsberg Depression Rating Scale (MADRS) [28]. Remission was defined as a MADRS score of ≤10 [29]. Response was defined as ≥50% reduction in MADRS score [29]. The clinician-rated cognitive function was assessed using the Mini-Mental State Examination (MMSE) [30]. Cognitive decline was defined as a reduction in MMSE of ≥2 points [31]. For patients missing pretreatment MMSE score but having a score registered within a maximum of two ECT treatment sessions after start of treatment, this adjacent score was imputed as the pretreatment score. Accordingly, if there was a missing posttreatment score of MADRS or MMSE, but an available score a maximum of two ECT treatment sessions before this point, this was imputed as the posttreatment score. Imputation applied to a limited number of scores. An overview of all imputed scores can be found in Supplementary Table 1.

### Patient-rated outcome measures

The self-rated depression severity was assessed using the 21-item Beck Depression Inventory (BDI) [32]. Remission was defined as BDI ≤ 9 [33], response as ≥50% reduction in BDI score [33]. For patients missing a pre- or posttreatment BDI score but having a score registered within a maximum of two ECT treatment sessions after (pretreatment) or before (posttreatment), this score was imputed (*n* = 3 pre and *n* = 7 posttreatment). Patients rated their cognitive function using the Everyday Memory Questionnaire (EMQ) [34], a 28-item questionnaire addressing practical, everyday memory functions. Cognitive decline was defined as an increase in EMQ-score exceeding 10%

### Statistics

Statistical analyses were conducted in SPSS 29.0 (IBM Corporation, Armonk, NY). Paired samples *t*-tests were used to investigate the change in symptom scores, differences between pairs were tested and the normality assumption was met. Logistic regression was used to identify predictors for achieving remission or experiencing cognitive decline. Selection of predictors was made based on clinical practice and previous literature. The regression model for remission included the predictors sex, age, having previously received ECT, duration of current episode, diagnosis of bipolar disorder, depression with psychotic features, the use of an antidepressant, antipsychotic (AP), anticonvulsant, benzodiazepine or z-hypnotic, and pretreatment MADRS/BDI level. The MADRS model additionally included psychiatric family history and age at the debut of affective symptoms, due to higher number of cases. Sex, age, previously received ECT, psychotic features, and pretreatment level of MMSE/EMQ were included as predictors in the models for cognitive decline. The number of predictors included in the models was limited to one for every 10 events, however, for some models, this limit was slightly exceeded [35]. Alpha level was set to 0.05; all tests were two-tailed. We used Cohen’s *d* to quantify the effect sizes, with interpretation for small effect size *d* = 0.2, medium effect size *d* = 0.5, and large effect size *d* = 0.8 [36].

### Ethics

Included patients provided written consent to the Regional Register for Neurostimulation Treatment in Western Norway, approved by the Norwegian Data Protection Authority (approval no. 2012/5490). The study was approved by the Regional Committee for Research Ethics (REK Nord, reference 2018/2541). ClinicalTrials.gov identifier (NCT number): NCT05388461.

## Results

The baseline demographic and clinical characteristics for the 392 included patients are shown in Table 1. The mean number of treatment sessions was 10.4 (SD 4.1).

**Table 1.** Clinical and demographical characteristics of 392 patients from the regional register for neurostimulation treatment in western Norway receiving ECT for depression

Characteristics		
Sex, female, *n* (%)	238	(60.7)
Age in years, *M* (SD)	54.8	(17.6)
Previously received ECT, *n (%)*	90	(23.0)
Age at debut affective symptoms, *M* (SD)	35.7	(19.5)
Family history of severe psychiatric disorders^a^, *n* (%)	219	(63.1)
Duration of current episode (weeks), *M* (SD)	31.2	(56.9)
Bipolar disorder, *n* (%)	83	(21.2)
Depression severity^b^		
Moderate, *n* (%)	57	(14.5)
Severe without psychotic features, *n* (%)	215	(54.8)
Severe with psychotic features, *n* (%)	120	(30.6)
Medication at first ECT-session^c^		
Antidepressant, *n* (%)	275	(70.2)
Antipsychotic, *n* (%)	251	(64.0)
Benzodiazepine/z-drug, *n* (%)	69	(17.6)
Anticonvulsant, *n* (%)	22	(5.6)
Lithium, *n* (%)	24	(6.1)
No psychotropic medication, *n* (%)	43	(11.0)

Abbreviation: ECT, electroconvulsive therapy.

*Note:* Percentages reported are valid percent.

a History of first- or second-degree relatives suffering from severe depression, bipolar disorder, or schizophrenia.

b Based on registered diagnosis, including both uni- and bipolar depression.

c Administered on a regular basis.

### Clinician-rated outcome measures

#### Depressive and cognitive symptoms pre/post ECT

Changes in depression symptom severity and cognitive function from pre- to posttreatment combined with results from paired *t*-test are shown in Table 2. There was a significant decrease in MADRS score, with large effect size. Clinician-rated remission rate (*n* = 384) was 49.5%. The MMSE score improved, however with a small effect size.

**Table 2.** Depressive symptoms and cognitive function pre and post ECT combined with statistics from paired *t*-tests comparing depressive symptoms and cognitive function pre and post ECT

			Pre	Post			
		*n*	*M* (SD)	*M* (SD)	*t*	*p*	Cohen’s *d*
Clinician rated	MADRS	374	35.4 (6.7)	12.0 (8.5)	40.75	<0.001	2.11
	MMSE	352	27.7 (2.7)	28.1 (2.3)	−2.83	0.005	0.15
Patient rated	BDI	296	33.7 (11.0)	14.8 (11.4)	24.83	<0.001	1.44
	EMQ	205	101.7 (43.1)	88.7 (39.1)	4.08	<0.001	0.29

Abbreviations: BDI, Beck Depression Inventory; ECT, electroconvulsive therapy; EMQ, Everyday Memory Questionnaire; MADRS, Montgomery-Åsberg Depression Rating Scale; MMSE, Mini-Mental State Examination.

A total of 348 patients had scores for both MADRS and MMSE. The categorization of patients according to clinician-rated remission status and cognitive decline in a four-quadrant model is shown in Table 3A. Nearly half of patients reached the remission criteria. In more than 85% of patients, there was no cognitive decline. A total of 43.7% were in the most favorable group of remission without cognitive decline. An overview of outcome variables, including missing data, is found in Supplementary Table 2A/B.

**Table 3.** Combinations of remission status and cognitive decline after ECT for depression in a four-quadrant model

A. Clinician-rated outcome measures			
Clinician-rated (*n* = 348)		Remission^a^	
		Yes (*n* (%))	No (*n* (%))
Cognitive decline^b^	No	152 (43.7)	148 (42.5)
	Yes	19 (5.5)	29 (8.3)

B. Patient-rated outcome measures			
Patient-rated(*n* = 198)		Remission^c^	
		Yes (*n* (%))	No (*n* (%))
Cognitive decline^d^	No	65 (32.8)	80 (40.4)
	Yes	9 (4.5)	44 (22.2)

Abbreviation: ECT, electroconvulsive therapy.

*Note:* Including patients with an available score for both outcome measures. Percentages reported are valid percent.

a Montgomery-Åsberg Depression Rating Scale ≤10.

b Mini-Mental State Examination reduction ≥2 points pre−/post ECT.

c Beck Depression Inventory ≤9.

d Increase in Everyday Memory Questionnaire score > 10% pre−/post ECT.

**Figure 1.**
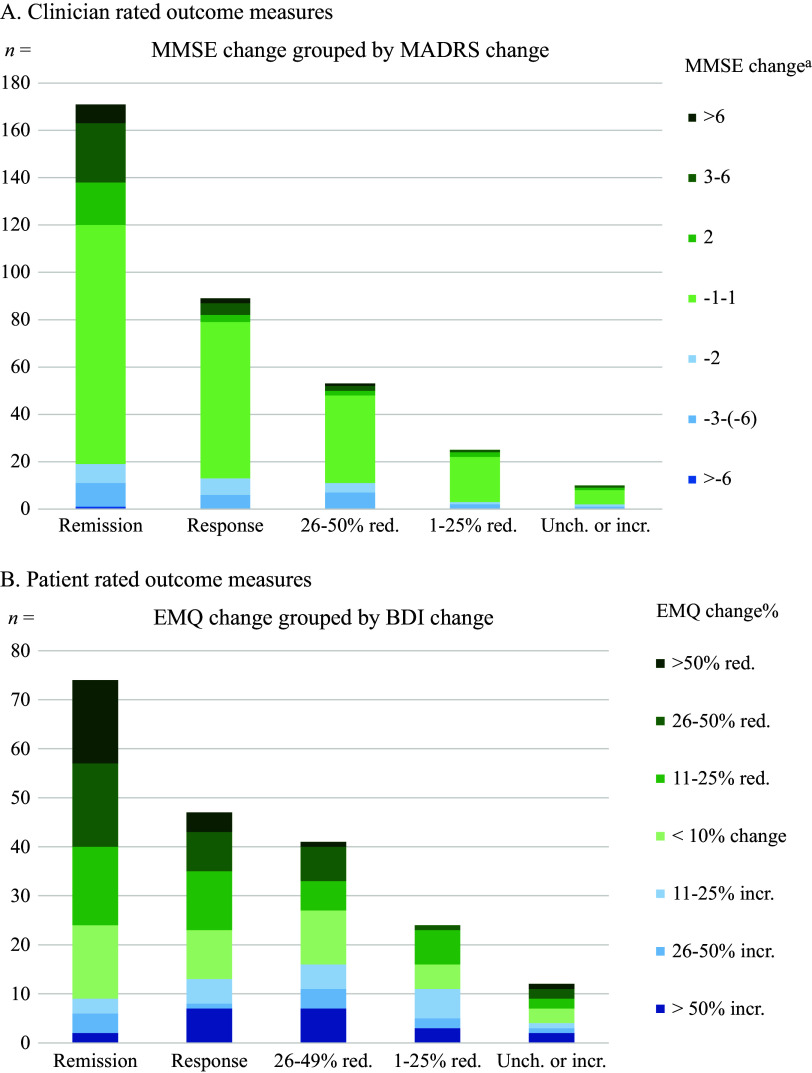
Cognitive change grouped by remission status and change in depression after ECT. Missing data are not included in the figures but can be found in Supplementary Table 1. (A) MMSE change in points grouped by remission status and MADRS change after ECT. Remission = MADRS ≤10; Response = ≥ 50% reduction MADRS score, but no remission. ^a^Change in MMSE in points pre/post ECT. A positive number equals an improvement in cognitive function. ECT, electroconvulsive therapy; incr., increase; MADRS, Montgomery-Åsberg Depression Rating Scale; MMSE, Mini-Mental State Examination; red., reduction; unch., unchanged. (B) EMQ change in percent grouped by remission status and BDI change after ECT. A decrease in EMQ score equals an improvement in cognitive function. Remission = BDI ≤ 9; Response = ≥ 50% reduction in BDI-score, but no remission. BDI, Beck Depression Inventory; ECT, electroconvulsive therapy; EMQ, Everyday Memory Questionnaire; incr., increase; red., reduction.

To further differentiate the combined depressive and cognitive outcome, we divided patients into more clinically relevant subgroups, depicted in a bar chart (Figure 1A) based on a 5 × 7 matrix (Supplementary Table 2A). In the group achieving remission, 152 (88.9%) patients had an unchanged or improved cognitive function. Conversely, in the subgroup of non-responders, 81.8% had an unchanged or improved cognitive function, leaving a group of 16 patients displaying unsatisfying effect on depressive symptoms in combination with cognitive decline. Of the total amount of patients with an available score for both clinician-rated outcome measures, this constitutes 4.6%.

#### Predictors for clinician-rated remission

We initially sought to identify predictors for being in the least favorable group with no effect on depressive symptoms in combination with cognitive decline after ECT. However, the small group sizes limited the number of predictors we could include. We therefore decided to do separate regression models for remission and cognitive decline. For all regression analyses, preliminary analysis suggested that the assumption of no multicollinearity was met. Results from the analysis for achieving remission are shown in Table 4. After introducing 14 predictors, 305 patients were included in the analysis for clinician-rated remission. The strongest predictors for achieving remission were using AP at first treatment (OR 3.04, *p* < 0.001) and previously having received ECT (OR 2.29, *p* = 0.020). Increasing age and psychotic features also increased the odds of achieving remission, whereas longer duration of depressive episode decreased the odds of remission. Sensitivity analyses were performed for findings regarding sex and AP (Supplementary Tables 3 and 4). These predictors were separately included in regression models including age, mean charge delivered, mean pulse width, and electrode placement. The use of AP remained significant whereas sex did not.

**Table 4.** Logistic regression predictors clinician and patient-rated remission after ECT

	MADRS^a^ (*n* = 305)			BDI^b^ (*n* = 280)		
	Exp. (*B*)	CI Exp. (*B*)	*p*	Exp. (*B*)	CI Exp. (*B*)	*p*
Sex	0.55	0.32–0.95	0.031	0.65	0.37–1.14	0.130
Age	1.03	1.01–1.05	0.011	1.03	1.02–1.05	< 0.001
Psychiatric family history^c^ ^,^^d^	0.95	0.54–1.67	0.856			
Age debut affective symptoms^d^	1.01	1.00–1.03	0.122			
Previously received ECT	2.29	1.14–4.61	0.020	0.94	0.47–1.88	0.854
Duration current episode^e^	0.99	0.97–1.00	0.006	0.99	0.98–1.00	0.038
Bipolar disorder	0.56	0.27–1.13	0.103	0.67	0.32–1.43	0.301
Psychotic features	1.99	1.07–3.70	0.030	3.33	1.78–6.22	< 0.001
Pretreatment MADRS/BDI	1.01	0.97–1.06	0.596	0.97	0.94–0.99	0.010
Antidepressant^f^	0.89	0.47-1.66	0.704	0.83	0.44–1.56	0.564
Antipsychotic^f^	3.04	1.68-5.50	< 0.001	1.74	0.96–3.17	0.070
Benzodiazepine/z-hypnotic^f^	0.86	0.41-1.81	0.688	0.98	0.47–2.04	0.958
Anticonvulsant^f^	0.86	0.28-2.69	0.796	1.90	0.61–5.90	0.271
Lithium^f^	2.83	0.91-8.83	0.073	1.39	0.36–5.37	0.635

Abbreviations: BDI, Beck Depression Inventory; ECT, electroconvulsive therapy; MADRS, Montgomery-Åsberg Depression Rating Scale.

a Remission = MADRS ≤10. Model statistically significant, *χ* ^2^ (14, *n* = 305) = 87.81, *p* < 0.001.

b Remission = BDI ≤ 9. Model statistically significant, *χ* ^2^ (12, *n* = 280) = 63.37, *p* < 0.001.

c History of first- or second-degree relatives suffering from severe depression, bipolar disorder, or schizophrenia.

d Predictor not included in the BDI regression analysis.

e Duration in weeks.

f Administered on a regular basis.

#### Predictors for clinician-rated cognitive decline

Results from regression analysis for cognitive decline (MMSE reduction ≥2) are shown in Table 5. Higher pretreatment MMSE score made cognitive decline more likely, OR 1.35 (*p* = 0.001). Male sex decreased the likelihood of displaying cognitive decline. In a sensitivity analysis including sex, age, mean charge delivered, mean pulse width and electrode placement (Supplementary Table 5), the effect of sex was no longer significant.

**Table 5.** Logistic regression predictors clinician and patient-rated cognitive decline after ECT

		MMSE^a^ (*n* = 352)			EMQ^b^ (*n* = 205)	
	Exp. (*B*)	CI Exp. (*B*)	*p*	Exp. (*B*)	CI Exp. (*B*)	*p*
Sex	0.50	0.26–1.00	0.049	1.12	0.55–2.27	0.751
Age	1.02	1.00–1.03	0.126	0.97	0.95–0.99	0.002
Previously received ECT	1.18	0.56–2.52	0.664	1.64	0.66–4.10	0.286
Psychotic features	1.03	0.49–2.14	0.939	0.35	0.14–0.88	0.025
Pre-treatment MMSE/EMQ	1.35	1.13–1.63	0.001	0.97	0.96–0.98	< 0.001

Abbreviations: ECT, electroconvulsive therapy; EMQ, Everyday Memory Questionnaire; MMSE, Mini-Mental State Examination.

a Cognitive decline defined as MMSE reduction ≥2 points pre topost ECT. Model statistically significant, *χ* ^2^ (5, *n* = 352) = 17.96, *p* = 0.003.

b Cognitive decline defined as EMQ increase >10% pre to post ECT. Model statistically significant, *χ2* (5, *n* = 205) = 42.95, *p* < 0.001.

### Patient-rated outcome measures

#### Depressive and cognitive symptoms pre/post ECT

There was a significant decrease in BDI from pre- to posttreatment, with large effect size (Table 2). Patient-rated remission rate (*n* = 349) was 41.0%. The EMQ decreased, indicating subjectively improved cognition after ECT, with a small effect size. There were 198 patients with available scores for both patient-rated outcome measures. Grouping of patient-rated cognitive symptoms by remission status in a four-quadrant model is shown in Table 3B, a bar chart based on a 5 × 7 matrix (Supplementary Table 2B) for BDI and EMQ change is shown in Figure 1B. Of patients subjectively achieving remission, 65 (87.8%) patients experienced unchanged or improved everyday memory. In the group of non-responders, 59.7% experienced an unchanged or improved everyday memory, leaving 31 patients with subjective non-response in combination with decline of experienced everyday memory, amounting to 15.7% of the total of 198 patients.

#### Predictors for patient-rated remission

For BDI, 12 predictors were included, leaving 280 patients in the analysis (Table 4). Increasing age and depression with psychotic features increased the odds of achieving remission, psychotic features being the strongest predictor, OR 3.33 (*p* < 0.001). Longer duration of the current episode and higher pretreatment depression level gave lower odds for achieving patient-rated remission.

#### Predictors for patient-rated cognitive decline

In the regression analysis for EMQ, 205 patients were included (Table 5). Depression with psychotic features decreased the odds of cognitive decline, OR 0.35 (*p* = 0.025), as did an increase in age and a higher level of pretreatment subjective everyday memory concerns. Sensitivity analyses were performed with alternative cut-offs for cognitive decline at 5 and 15%, respectively. Cut-off at 5% yielded the same significant predictors as in the original analysis. With cut-off at 15%, psychotic features were no longer a significant predictor (*p* = 0.074), but the OR of 0.41 was only slightly different from the original analyses (OR 0.35). In summary, results from the main model and the two sensitivity models were consistent, indicating the robustness of findings in the main model.

## Discussion

In this register-based study, we investigated the effects of ECT on depressive symptoms and cognitive function in a routine clinical setting, and predictors for achieving remission and cognitive decline. Our study is, to our knowledge, the first to present detailed matrices combining outcomes on mood and cognition. Our main finding was that for most patients, ECT was effective with no deleterious effect on cognition. However, there was a noteworthy group of patients experiencing unsatisfactory effect on depressive symptoms in combination with cognitive decline. Several predictors for achieving remission or experiencing cognitive decline were identified.

The basis of an informed consent is that patients are provided with comprehensive, adequate, and valid information about potential benefits and risks of a treatment. It is of essence that patients do not experience coercion [37], particularly as patients can be vulnerable and their ability to make a decision can be affected by their disease. While the efficacy of ECT is well established, effects on different domains of cognition remains to be fully elucidated, and the balance between ethical principles like autonomy, beneficence, and non-maleficence needs to be taken into consideration when discussing the treatment. Continuous evaluation of ECT through research on efficacy and effectiveness, side effects, and mechanisms of action is important to reduce stigma and provide patients and caretakers with balanced and transparent information about this important psychiatric medical procedure.

### Effects on mood

Remission is considered an important goal in depression treatment [38]. Our study revealed lower remission rates (41.0% patient-rated and 49.5% clinician-rated), compared to clinical trials (70–90%) [23, 39]. Similar findings are seen in research from community settings [21], and in the Swedish National Quality Registry for ECT (self-rated remission 42.8%) [40]. Discrepancies may be due to factors such as stricter selection criteria, closer monitoring of patients, different level of resources, less variability in application of ECT and more complete assessments in clinical trials.

A main goal of our study was to provide additional information on predictors of outcome to patients and clinicians in their process of deciding on ECT. Prior research has demonstrated an association between higher age and response and remission after ECT [29, 40–42], our findings confirmed that increasing age was a positive predictor for achieving remission. It has been suggested that this effect is mediated by the presence of psychomotoric disturbances and psychotic symptoms [43, 44].

While a meta-analysis by Haq et al. considered the presence of psychotic symptoms less likely to be clinically useful due to only a weak association [45], our findings align with recent meta-analyses [29] and reviews [41], indicating that depression with psychotic features predicts a favorable outcome after ECT. Having previously received ECT predicted clinician-rated remission, aligning with previous research [46]. This is probably explained by a positive outcome from ECT in the past increasing the chances of choosing ECT again. We also found that a longer duration of the depressive episode predicted poorer outcome, an association seen with other treatments as well [47], and consistent with previous ECT-studies [45, 48]. This raises concern, given that ECT typically is recommended as a last-resort treatment.

A higher level of subjective depression score before ECT predicted lower odds for achieving remission. To our knowledge, the association between pretreatment self-assessed depression level and subjective remission, has not previously been investigated. A Swedish registry study investigated predictors for achieving self-assessed remission, including pretreatment Clinical Global Impression Severity Scale as a predictor [40]. They found a tendency toward higher subjective remission rates in the more severely ill patients, but this association was not significant after adjustments in their statistical model [40]. They did find, however, that several psychiatric comorbidities were associated with lower remission rates, calling for further investigation to explore the potential influence of comorbidities.

A more surprising finding was that the use of AP was associated with higher odds for achieving clinician-rated remission, psychotic features being adjusted for in the model. Sensitivity analysis including age and treatment parameters did not alter this finding (Supplementary Table 3). One explanation could be that AP lower ST and thus yield a more efficient seizure, albeit findings concerning psychotropic medication and its effects on ST are diverging [49]. Another possibility could be that clinicians use AP to treat psychotic depression even without making the diagnosis. As AP are included in treatment algorithms for uni- and bipolar depression, this is an interesting finding warranting further investigation.

### Effects on cognition

Although not developed for this purpose, the MMSE is widely used to monitor global cognitive function in an ECT-setting [17]. It is limited by ceiling effect, not being sensitive to more subtle changes, and not addressing executive functions [18, 31, 50]. Further, in our clinical setting, the clinicians are encouraged to assess cognitive function on a weekly basis, representing a risk for practice effects, although usually alternate forms of some items are used. We found a group of 13.6% of patients displaying cognitive decline (MMSE reduction ≥2 points). On a group level, there was a significant improvement in MMSE pre to post-ECT, in line with previous studies [50]. Others, including Semkovska and McLoughlin in their meta-analysis, have found a significant decline in MMSE shortly after ECT, before regaining pretreatment scores [17]. Possible explanations for these differences could be time of assessment, patient selection, and their cognitive status pre-ECT. It is well established that depression itself has negative effects on cognition [51], and the relief of depressive symptoms will potentially lead to cognitive improvement [52]. Our finding that a lower MMSE score pre-ECT predicts lower odds for displaying cognitive decline, may be due to the impact of depression on baseline MMSE scores and subsequent relief of depression leading to cognitive improvement, in addition to lower pre-MMSE score making practice effects more lightly [50]. The MMSE also exhibits a ceiling effect, leading to the only possibility for many patients being either a reduction in, or an unchanged, score. In summary, this finding should be interpreted with caution.

Although there is no consensus on which cognitive assessments to use in an ECT-setting, the importance of including subjective cognitive instruments has been emphasized [53]. Most neuropsychological tests are designed to address specific cognitive aspects, and may not capture the complexities of everyday memory functioning, their ecological validity has been questioned [54]. The EMQ was originally developed for assessing everyday memory in patients with traumatic brain injuries but has also previously been used in an ECT-setting [55, 56]. Although the change in everyday memory scores on a group level indicated a small improvement, we found that a group of 26.3% of patients experienced a worsening of subjective cognition. There is, to our knowledge, no previous literature on a specific cut-off value for cognitive decline in the EMQ. We defined the cut-off for subjective cognitive decline quite strict, reducing the risk of not capturing patients with mentionable deterioration. We also performed sensitivity analysis to add robustness to the cut-off criterion, as described in the results section. Our finding is in line with results from a similar Swedish population, finding 26% experiencing subjective memory worsening (defined as 2 point drop on a 7-point Likert scale) [57]. In a recent review and meta-analysis, however, the weighted mean of subjective cognitive complaints was 48.1% [58]. Studies on subjective effects on cognition have found divergent results [20, 58], possibly due to different assessment scales and cut-off scores, as well as the degree of post-ECT residual depressive symptoms [58].

We found that higher age, depression with psychotic features and higher pretreatment level of EMQ predicted lower odds for patient-rated cognitive decline. Younger patients with less pretreatment subjective memory problems had higher risk for subjective memory worsening, in line with a Swedish register-based study [57]. The same Swedish study did not identify psychotic features as a significant predictor, possibly due to them including post-ECT depression scores and treatment parameters in the model [57]. An association between younger age and more subjective than objective adverse effects from pre- to posttreatment was found by Hammershøj et al. in a study of 41 unipolar and bipolar depressed patients undergoing BL ECT [19]. Possible explanations for their finding included that young depressed people display less adaptive cognitive coping mechanisms, and have better access to information about possible adverse effects after ECT, possibly negatively affecting their expectations [19]. Negative expectations have been associated with a worsening in subjective memory after ECT [59].

### Depressive symptoms and cognitive function beyond group levels

As for all treatments, results on a group level encompass a range of patient outcomes, spanning from deterioration to improvement in both depressive and cognitive symptoms. Presenting results beyond group levels has been called for [60]. Although our results showed a significant improvement in both clinician and patient-rated depression and cognition scores, with most patients responding without cognitive decline, the results also acknowledge and depict that some patients had a less desirable outcome. Transparency regarding this is important in the process of giving patients balanced information about this still controversial treatment. Finally, it is essential for clinicians to consider both clinician and patient-rated measures when evaluating the outcome of ECT [53, 61].

### Strengths and limitations

The strength of this study was the large sample with good ecological validity. However, approximately 15% of patients receiving ECT in our region did not consent to inclusion in the register; we cannot exclude the possibility of this subgroup representing a different symptom profile. In addition, there were missing data, especially for subjective assessments, reducing the generalizability of our results. Due to the nature of registry studies, the time of psychometric and cognitive assessments, did vary. This could potentially influence our results in either direction, for example, assessments done prior to end of treatment could possibly underestimate reduction in depressive symptoms and overestimate cognitive function. Diagnoses were provided by treating clinicians but were not confirmed by standardized diagnostic instruments. We did not have information on level of education or premorbid IQ-level, and so we were not able to adjust for these factors in the statistical analysis. As there is no consensus on which instruments to use [62, 63], the registry does not include data on retrograde autobiographical memory, although this is of great concern for patients [64]. Our study reports findings after acute ECT-treatment; it would clearly be of interest to assess long-term outcomes after ECT, with the same methodology.

## Conclusion and clinical implications

Our study supports ECT as an effective and safe treatment for depression. ECT should be considered at an early stage for older patients suffering from a depression with psychotic features, as this group seems to have a more favorable outcome. Existing literature suggests that patients treated with ECT feel that they are inadequately informed about different aspects of the treatment [65]. The findings from our study combining clinician-rated and patient-rated outcomes both on effectiveness and cognitive function are relevant for the education of patients and their families. Having access to results revealing the diversity in patients’ outcomes, will be of additional use for patients when deciding on treatment options. Our study contributes to the existing literature on predictors of effectiveness and cognitive decline following ECT. Identifying such predictors is of importance in foreseeing which patients that are most likely to benefit from the treatment, and moreover, identifying which patients that require closer monitoring. Further establishing such clinical predictors may also be of importance for the development of future biological predictors [45].

## Supporting information

Sellevåg et al. supplementary material Sellevåg et al. supplementary material

## Supplementary material

The supplementary material for this article can be found at https://doi.org/10.1192/j.eurpsy.2024.1.
