# Supplementary material for: Unmasking patient diversity: Exploring cognitive and antidepressive effects of electroconvulsive therapy
Source: Eur Psychiatry. 2024 Jan 12;67(1):e12. doi: 10.1192/j.eurpsy.2024.1 (PMC10964271; doi:10.1192/j.eurpsy.2024.1)
Supplement: Sellevåg et al. supplementary material [file S0924933824000014sup001.docx]

**Supplementary material**

**Table 1**

*Overview of Number of Imputed Scores Pre and Post ECT*

| Pre Post  *n* = *n* = |
| --- |
| MADRS 0 4  MMSE 4 11  BDI 3 7 |

*Note.* Abbreviations: ECT = Electroconvulsive therapy; MADRS = Montgomery-Åsberg Depression Rating Scale; MMSE = Mini-Mental State Examination; BDI = Beck Depression Inventory

**Table 2**

*Change in MMSE/EMQ pre-/post ECT Grouped by Remission, Response and Change in MADRS/BDI, Including Missing Data*

1. MADRS and MMSE

|  |  |  | MMSE change in points | | |  |  |  |
| --- | --- | --- | --- | --- | --- | --- | --- | --- |
|  | <-6 | -3 to -6 | -2 | -1-1 | 2 | 3-6 | >6 | Missing |
| Remission^a^ | 1 | 10 | 8 | 101 | 18 | 25 | 8 | 19 |
| Response^b^ | 0 | 6 | 7 | 66 | 3 | 5 | 2 | 10 |
| 26-49% red. | 0 | 7 | 4 | 37 | 2 | 2 | 1 | 2 |
| 1-25% red. | 0 | 2 | 1 | 19 | 2 | 1 | 0 | 1 |
| Unch.or incr. | 0 | 1 | 1 | 6 | 1 | 1 | 0 | 0 |
| Missing | 0 | 0 | 0 | 1 | 0 | 2 | 1 | 8 |

1. BDI and EMQ

|  |  |  | EMQ change in % | | |  |  |  |
| --- | --- | --- | --- | --- | --- | --- | --- | --- |
|  | > 50% incr. | 26-50% incr. | 11-25% incr. | <10% change | 11-25% red. | 26-50% red. | >50% red. | Missing |
| Remission^c^ | 2 | 4 | 3 | 15 | 16 | 17 | 17 | 69 |
| Response^d^ | 7 | 1 | 5 | 10 | 12 | 8 | 4 | 23 |
| 26-49% red. | 7 | 4 | 5 | 11 | 6 | 7 | 1 | 20 |
| 1-25% red. | 3 | 2 | 6 | 5 | 7 | 1 | 0 | 14 |
| Unch.or incr. | 2 | 1 | 1 | 3 | 2 | 2 | 1 | 5 |
| Missing | 1 | 0 | 0 | 1 | 1 | 2 | 2 | 56 |

*Note.* A. An increase in MMSE equals a cognitive improvement. ^a^MADRS ≤ 10. ^b^≥ 50% reduction in MADRS score pre-/post ECT.
B. An increase in EMQ equals cognitive decline. ^c^BDI ≤ 9. ^d^≥ 50% reduction in BDI score pre-/post ECT.
Abbreviations: MMSE = MiniMental Status Examination; EMQ = Everyday Memory Questionnaire; ECT = Electroconvulsive Therapy; MADRS = Montgomery-Åsberg Depression Rating Scale; BDI = Beck Depression Inventory; red. = reduction; unch. = unchanged; incr. = increase.

**Table 3**

*Sensitivity Analysis (Logistic Regression) Sex Predicting Remission^a^ After ECT, Including Age and Treatment Parameters*

|  |  | n = 382 |  |
| --- | --- | --- | --- |
|  | *Exp. (B)* | *CI Exp. (B)* | *p* |
| Sex | 0.82 | 0.51-1.32 | 0.408 |
| Age | 1.07 | 1.04-1.10 | < 0.001 |
| Mean charge delivered | 1.00 | 0.99-1.00 | 0.020 |
| Mean impulse width | 1.38 | 0.02-87.71 | 0.879 |
| Electrode placement RUL (ref.) |  |  |  |
| Electrode placement BL | 0.19 | 0.04-0.91 | 0.037 |
| *Note.* ^a^Remission = MADRS ≤ 10.  Abbreviations: ECT = Electroconvulsive therapy; RUL = Right unilateral electrode placement; BL= Bilateral electrode placement. | | |  |

**Table 4**

*Sensitivity Analysis (Logistic Regression) Use of an Antipsychotic Predicting Remission^a^ After ECT, Including Age and Treatment Parameters*

|  |  | n = 382 |  |
| --- | --- | --- | --- |
|  | *Exp. (B)* | *CI Exp. (B)* | *p* |
| Antipsychotic^b^ | 1.87 | 1.19-2.96 | 0.007 |
| Age | 1.07 | 1.04-1.10 | < 0.001 |
| Mean charge delivered | 1.00 | 0.99-1.00 | 0.015 |
| Mean impulse width | 1.11 | 0.02-75.19 | 0.960 |
| Electrode placement RUL (ref.) |  |  |  |
| Electrode placement BL | 0.16 | 0.03-0.76 | 0.021 |
| *Note.* ^a^Remission = MADRS ≤ 10.  ^b^Registered at first treatment session, administred on a regular basis.  Abbreviations: ECT = Electroconvulsive therapy; RUL = Right unilateral electrode placement; BL= Bilateral electrode placement. | | |  |

**Table 5**

*Sensitivity Analysis (Logistic Regression) Sex Predicting Cognitive Decline^a^ After ECT, Including Age and Treatment Parameters*

|  |  | *n* = 350 |  |
| --- | --- | --- | --- |
|  | *Exp. (B)* | *CI Exp. (B)* | *p* |
| Sex | 0.57 | 0.28-1.17 | 0.126 |
| Age | 1.00 | 0.97-1.04 | 0.801 |
| Mean charge delivered | 1.00 | 1.00-1-00 | 0.960 |
| Mean impulse width | 2.30 | 0.00-812.4 | 0.781 |
| Electrode placement RUL (ref.) |  |  |  |
| Electrode placement BL | 0.00 | 0.00- | 0.999 |
| *Note.* ^a^MMSE reduction ≥ 2 points pre-/post ECT.  ^b^Registered at first treatment session, administered on a regular basis.  Abbreviations: ECT = Electroconvulsive therapy; MMSE = Mini Mental State Examination; RUL = Right unilateral electrode placement; BL= Bilateral electrode placement. | | |  |
